# Editorial: The paracannabinoid system: endocannabinoid-like lipids and their functions

**DOI:** 10.3389/fendo.2023.1263924

**Published:** 2023-11-01

**Authors:** Maria Beatrice Passani, Gustavo Provensi, Daniele Piomelli

**Affiliations:** ^1^ Department of Health Sciences, University of Florence, Florence, Italy; ^2^ Department of Neuroscience, Psychology, Drug Research and Child Care University of Florence, Florence, Italy; ^3^ Department of Anatomy and Neurobiology, University of California, Irvine, Irvine, CA, United States; ^4^ Department of Biological Chemistry, University of California, Irvine, Irvine, CA, United States; ^5^ Department of Pharmaceutical Sciences, University of California, Irvine, Irvine, CA, United States

**Keywords:** fatty acyl ethanolamides, intestinal microbiota, oleic acid, maternal high fat diet, vagal sensory fibres

This Research Topic of *Frontiers in Endocrinology* features a series of studies focused on the paracannabinoids, a family of lipid-derived signalling molecules that include fatty acyl ethanolamides (FAEs) like oleoylethanolamide (OEA) and palmitoylethanolamide (PEA), and fatty acyl esters, like 2-oleoyl-*sn*-glycerol. In contrast to the endocannabinoids, paracannabinoid substances are independent of CB_1_ or CB_2_ receptors; their actions require the activation of nuclear receptors such as peroxisome proliferator-activated receptor-α (PPAR-α), cell-surface transient receptor potential cation channel vanilloid-1 (TRPV1), and G- protein coupled receptor 119 (GPR119). However, most of the documented biological effects of OEA and PEA are exerted through PPAR-α ligation, which may account for their anti- inflammatory action, the control of energy metabolism (11700558), suppression of peripheral nociception [9685157, 24473264], and neuroinflammation (35176443).

The paracannabinoid signalling system contributes to numerous physiological processes in various organs, where it participates in the control of intestinal motility (16143133), lipolysis in white adipose tissue (15123613, 35214069), ketone body formation in the liver, which requires activation of H1 histamine receptors [30318340). Paracannabinoids most often operate in concert with the endocannabinoid system, providing parallel or antagonistic regulatory roles. A classic example is how endocannabinoids and paracannabinoid levels in the gastrointestinal tract change in opposing fashion according to the nutritional status. They also display opposite physiological effects along the gut-brain axis, where anandamide stimulates food intake whereas OEA suppresses it (12417686, 11700558).

In the small intestine, dietary oleic acid stimulates OEA production (23567058, 11700558) and in turn, OEA is metabolized into oleic acid and ethanolamine via two enzymatic pathways, the fatty acid amide hydrolase which is expressed in a variety of tissues, and the N- acylethanolamine hydrolysing acid amidase expressed in the intestinal epithelium (17121838). In this Research Topic, Igarashi et al. posited that dietary oleic acid is an important satiety-inducing molecule. By feeding mice with a low-content or control oleic acid diet for several weeks, the authors show the impact of dietary oleic acid in the regulation of food intake and satiety via intestinal sensing of dietary fat and OEA, excluding the influence of other FAEs such as PEA and linoleoyl-ethanolamide. Studies in human subjects have shown that levels of dietary oleic acid or intraduodenal oleic acid infusion reduce appetite and energy intake (25347552, 21310831), therefore the study by Igarashi et al. strengthens the notion of nutritional balance as a primary regulator of energy consumption.

Very few studies have investigated the role of endo- and paracannabinoids during lactation, and how these may shape the offspring’s nutrient choice and metabolic profile. For instance, there is a negative correlation between milk contents of OEA, PEA and stearoylethanolamide, and infants’ body weight during lactation (30428553). Maternal hypo- and hypercaloric diets change considerably the profile of both endo-and paracannabinoids in peripheral tissues as well as in the brain (27847471, 26778987). It is assumed that maternal high-fat diet during gestation and lactation, besides changing the endocannabinoid system in a tissue-specific manner, instructs obesity in rodents’ offspring (30776574). In this Research Topic, Dias-Rocha et al. show that maternal high-fat diet reduced anandamide and 2-arachidonoyl-*sn*-glycerol in rat breast milk, and increased adiposity in male and female offspring at weaning. Maternal high- fat diet also increased endocannabinoids and dopamine signalling in the nucleus accumbens of male offspring, a response associated with higher preference for fat in adolescence. The authors suggest that changes in the endocannabinoid pathway at weaning may contribute to the earlier metabolic phenotype in males, which present a more severe dysmetabolism in the long-term.

OEA is one of the gut-derived satiety factors relaying signals to the hypothalamus to regulate feeding (12955147, 25049422), and to other brain structures to strengthen memory consolidation (19416833, 28339575). In this respect, OEA is one of the key players in the complex communication between the periphery and the central nervous system, linking peripheral functions with cognitive and emotional brain centres. The path by which OEA conveys signals from the periphery to the brain is still a matter of debate. Early studies suggested sensory afferent fibres as the main route (11700558). In this Research Topic, Romano et al. consider the bloodstream as an alternative way for OEA to affect central homeostatic actions and cognitive responses. The authors report data suggesting that vagal sensory fibres may not be required for the neurochemical effects of exogenous OEA. After intraperitoneal administration, OEA concentrations increased in brain areas associated with the inhibition of food intake within few minutes, confirming that exogenously administered OEA can modulate eating behaviour (11700558), possibly by directly targeting neurons of specific brain nuclei. However, the fact that circulating levels of endogenous OEA do not increase after a meal (11700558) reminds us of the complexity of this problem and suggests that more work is needed to unravel it.

Dysregulation of the endocannabinoid system has been associated with pathological conditions such as obesity, type 2 diabetes, and intestinal inflammation, and there is convincing evidence that selected intestinal microorganisms and endocannabinoids covary in pathological conditions such as the ones mentioned above (32106469). The active interplay between microbiota and paracannabinoids as well is beginning to be acknowledged as a potent regulatory system of the gastrointestinal homeostasis. De Filippo et al. summarise in their review recent findings indicating a strict relationship between OEA and the intestinal microbiota in health and disease. The authors suggest that OEA acts as a *trait d’union* between gut microbiota and dynamic physiological and homeostatic processes. It is, therefore, conceivable that the malfunctioning of the crosstalk between paracannabinoids and intestinal microorganisms contributes to a variety of disorders such as enteropathies, obesity and associated chronic inflammatory status.

This Research Topic of Frontiers in Endocrinology highlighted differences and similarities between the endo- and paracannabinoids, and hopefully it will inspire further research to better understand the complex modes of action of these systems.

## Author contributions

MBP: Conceptualization, Writing – original draft, Writing – review & editing. DP: Conceptualization, Writing – original draft, Writing – review & editing. GP: Writing – original draft, Writing – review & editing.

